# DIETARY PROTEIN RESTRICTION IN AGED MALE MICE REDUCES MARKERS OF SASP IN ADIPOSE TISSUE

**DOI:** 10.1093/geroni/igae098.3651

**Published:** 2024-12-31

**Authors:** Molly Easter, Khristina Young, Hanna Sothers, Jolaiya Aldridge, Yvann Batamack, Prerana Vaddi, Christopher D Morrison, Cristal Hill

**Affiliations:** University of Southern California, Los Angeles, California, United States; University of Southern California, Los Angeles, California, United States; University of Alabama at Birmingham, Birmingham, Alabama, United States; University of Southern California, University of Southern California, California, United States; University of Southern California, Los Angeles, California, United States; University of Southern California, Leonard Davis School of Gerontology, Los Angeles, California, United States; Pennington Biomedical Research Center, Baton Rouge, Louisiana, United States; University of Southern California, Los Angeles, California, United States

## Abstract

Aging is associated with cellular senescence and metabolic impairment of adipose tissue. Moreover, the detriments of aging are further exacerbated by obesity on metabolic function in adipose tissue. Our lab and others have shown that dietary protein restriction (DPR), without reducing calorie intake, protects against age-related metabolic decline and improves lifespan. To determine if DPR reduces cellular senescence in adipose tissue during aging and aging with obesity, 16 month old male C57BL/6 mice were fed either a normal-protein (20%), low-protein (5%), high-fat (60% fat) or a low-protein/high fat (5% protein; 60%fat) diet for 24-weeks, then adipose depots were analyzed via real time quantitative PCR (RT-qPCR) for cellular senescence markers. We found significant decreases in p21, senescence associated secretory phenotype markers(SASP) and reduced SA-Bgal staining in adipose tissue of mice that were fed a low protein diet compared to normal protein diet fed mice. Additionally, mice on a low protein diet and high fat diet demonstrated significant decreases in cellular senescence markers compared to mice on a normal protein and high fat diet, suggesting that a low protein diet decreases the burden of cellular senescence on adipose tissue in aged mice and aged obese mice. Overall, these data suggests that DPR reduces the burden of cellular senescence on adipose tissue function during aging and aging with obesity. Given the negative impact of cellular senescence on adipose tissue, DPR offers a potential dietary intervention to prevent the detriments of cellular senescence on adipose tissue function during aging and aging with obesity.

